# The Making of Models Which Are Durable, and Which Can Be Painted

**Published:** 1902-01

**Authors:** William H. Potter


### Reviews of Dental Literature.

---

The Making of Models which are Durable, and which can be painted. By Dr. G. Port, Heidelberg,[1]

The ordinary model made from plaster of Paris is not as hard and durable as could be desired, and it fails to suggest the color of the tissues which it represents. The author takes it for granted that the production of a model more durable than the one commonly made, and also of one which can be painted so as to represent in a lifelike way the conditions of the tissues as they exist either in health or disease, would be an end much to be desired. And, first, he reviews the way in which attempts have been made for the improvement of models.

A much used method is to simply paint the plaster model with an oil color. The author says that this does not accomplish much, as the color soon strips off, and the appearance and value of the model are thus seriously impaired. A second method is that of Professor de Marion and Touvet-Fanton. By this method, plaster of Paris is abandoned and a mixture of two parts paraffin to one each of wax and stearin is used. These three substances are melted together and coloring-matter added. To reproduce the color of gum-tissue madder-red and carmine are used, and for the tooth-substance mignonette yellow. The properly colored waxy mixture is poured warm into the impression and a model produced. The

---

[1] Die Herstellung dauerhafter und bemalbarer Modelle, von Dr. med. G. Port, a. o. Universitätsprofessor und Leiter des zahnärztlicher Instituts in Heidelberg. Oesterreichisch-ungarische Vierteljahrsschrift für Zahnheilkunde, October, 1901.

process of making a model in this way is very complicated, and takes much time. Very beautiful results can, however, be obtained by it.

The author next speaks of a much used process whereby a plaster model is boiled in stearin. By this means a model obtains a very beautiful yellowish color, but its durability is not really increased. A fourth method is that of Julke. Take six parts of plaster of Paris and one part of freshly slacked lime. Mix these together and use as ordinary plaster. When the models are well dried, place in a ferrosulphate and zinc sulphate solution. A fifth method consists in immersing plaster models, which have been well dried, in an alum solution. The models to be immersed for half an hour. The solution should be made of one part iron-free alum and six parts water. Although much has been attained for the improvement of models by the above-mentioned methods, the author believes that his own method is a decided advance upon those described. He uses a mixture of plaster of Paris, chalk, and a glue solution. This combination is poured into the impression and allowed to harden. The glue to be used is not the common cabinet-maker's glue, but French hare or rabbit glue. Of this glue a three to five per cent. solution is made. After the preparation of the glue solution, a mixture of plaster and chalk is made in the proportion of three parts plaster and one part chalk. This mixture must be very exact. The plaster-chalk mixture must now be made with the glue solution into a stiff porridge. This porridge cannot be poured into an impression as if it were a simple plaster mixture; it is too viscous. A hair paint-brush must be used, and the material must be painted into the irregularities of the impression. By this means the impression can be filled. At least twelve hours are required for hardening before the model can be separated from the impression. Any form of impression material can be used. A model made in this way has at first almost the hardness of a plaster model, and after about eight days it is like hard wood to cut with a knife. The model must now be prepared for painting by a coating of linseed oil. When this is thoroughly dry, oil colors can be used. These must be much thinned with turpentine. It is better to apply several thin coats than one thick one. The author gives the following list of colors which he finds useful in the painting of models:

1. Madder lake 3, dark rose.
2. Bright, English red.

3. Carmine cinnabar.
4. Light ochre 1.
5. Clear, brilliant, yellow.
6. Terra di Siena.
7. Prussian blue.
8. Parisian, ultramarine.
9. Clear green, cinnabar.
10. Burned terra di Siena.
11. Ivory black.
12. Kremnitz white.

For the representation of normal mucous membrane a mixture of carmine cinnabar and Kremnitz white is to be used. Madder lake with white gives a rose-color shading into a blue which represents an inflamed mucous membrane. The color of the teeth is produced by a mixture of ochre and white. The unessential parts of the model are painted black. Models constructed by the above methods are not only very durable, but are also true to nature, and well repay the time spent upon them.

William H. Potter.

